# Percutaneous kyphoplasty versus posterior spinal fixation with vertebroplasty for treatment of Kümmell disease: A case–control study with minimal 2-year follow-up

**DOI:** 10.1097/MD.0000000000009287

**Published:** 2017-12-22

**Authors:** Hou-Kun Li, Ding-Jun Hao, Jun-Song Yang, Da-Geng Huang, Cheng-Cheng Yu, Jia-Nan Zhang, Lin Gao, Han Li, Bing Qian

**Affiliations:** Department of Spine Surgery, Honghui Hospital, Xi’an Jiaotong University, Xi’an, Shaanxi, China.

**Keywords:** Kümmell disease, percutaneous kyphoplasty, posterior fixation with vertebroplasty, spinal surgery

## Abstract

This is a retrospective case–control study.

The aim of this study was to compare the surgical results of percutaneous kyphoplasty (KP) and posterior spinal fixation with vertebroplasty (PSF+VP) for treatment of Kümmell disease (KD).

KD is rare form of post-traumatic delayed avascular necrosis of the vertebral body. It is reported that KP is an effect measure for treatment of KD. Some studies have recommended posterior spinal fixation with vertebroplasty for KD.

A total of 100 patients with KD who underwent spinal surgery at our hospital were enrolled from January 2008 to December 2013. The inclusion criteria were monosegment lesion without neurological deficit; the segments are restricted to T11-L2; conservative treatment is invalid. The exclusion criteria were metastatic spinal tumors, infection, primary bone tumor, and multiple myeloma; bisegments and multi-segments; patients with neurological symptoms; the defect of posterior wall of vertebral body; the occupying of vertebral canal. The symptomatic vertebrae were restricted to T11–L2. Patients who were followed-up for less than 2 years after surgery were excluded. Finally, there are 25 patients in the KP group and 21 in the PSF+VP group. There were no significant differences in patient age, disease duration, or the length of follow-up between the 2 groups.

Operative time (43.2 ± 21.8 vs 230.6 ± 87.1 minutes) was significantly longer and bleeding volume (5.3 ± 3.1 vs 215.0 ± 170.2 mL) significantly greater in the PSF+VP group. No significant difference between the 2 groups was observed in Visual analog scale score (VAS) (1.3 ± 0.9 vs 1.2 ± 0.9), Oswestry disability index score (ODI) (27.2 ± 9.0 vs 26.0 ± 6.3), and Cobb angle (17.0 ± 7.2 vs 16.5 ± 2.8). KP resulted in a shorter operation time, less bleeding volume, and fewer postoperative complications than PSF+VP.

This study shows that both treatments KP and PSF+VP for KD can be safe and effective for the patients with monosegment lesion and without neurological deficit. However, KP show the advantages in a shorter surgical duration, less blood loss, and fewer postoperative complications.

## 1 Introduction

Kümmell disease (KD) is becoming more common with the aging of the population. It was first described in 1895 by the German surgeon Hermann Kümmell as osteonecrosis of the vertebrae that typically occurs in the elderly in response to acute spinal trauma and often remains asymptomatic for several weeks or months, eventually resulting in clinical symptoms and kyphosis deformity.^[1–4]^ The main finding of diagnostic imaging of KD is characterized by a vacuum sign that surrounded by harden bone and it cannot self-heal.^[5–11]^ It is a rare form of post-traumatic delayed avascular necrosis of the vertebral body.

Spinal surgery for KD patients with invalid conservative treatment is inevitable. Various surgical procedures have been proposed in the management of KD. It is reported that percutaneous kyphoplasty (KP) is an effective measure for the treatment of KD.^[12]^ Some studies have recommended posterior spinal fixation with vertebroplasty for KD because of the lack of cement diffusion and bone cement displaced in this special condition.^[12–14]^

Up to now, only case reports and small series of patients have been reported. In 2011, Lee et al^[15]^ reported satisfactory results for 10 patients with KD treated by vertebroplasty combined with short segmental fixation. However, the sample of the patients are relatively small. It is unclear, however, which is actually preferable: KP—minimally invasive surgery using kyphoplasty or a surgical technique using posterior spinal fixation with vertebroplasty (PSF+VP).

As far as we know, there have been no reports to compare the 2 surgical results for KD with monosegment lesion without neurological deficit, and with minimal 2-year follow-up. The purpose of this study was to compare the mid-long-term surgical results and radiological outcomes of KP and PSF+VP for treating KD.

## 2 Material and methods

### 2.1 General date

This study was a retrospective case–control study. Institutional review board approval was obtained from our hospital for medical record review. A diagnosis of KD was based on a history of minor trauma and mild back pain and returned to normal life after a short period of rest and symptomatic treatment, and the radiographic examination, including progressive collapse of the vertebral fracture, intravertebral cleft, bone absorption, or sclerosis of the residual bone.

A total of 100 patients with KD who underwent spinal surgery at our hospital were enrolled from January 2008 to December 2013. All patients with vertebral fractures were treated conservatively without neurological deficits.

The inclusion criteria included monosegment lesion without neurological deficit; the segments are restricted to T11-L2; conservative treatment is invalid. The exclusion criteria included metastatic spinal tumors, infection, primary bone tumor, and multiple myeloma; bisegments and multi-segments; patients with neurological symptoms; the defect of posterior wall of vertebral body; and the occupying of vertebral canal.

**Figure 1:**
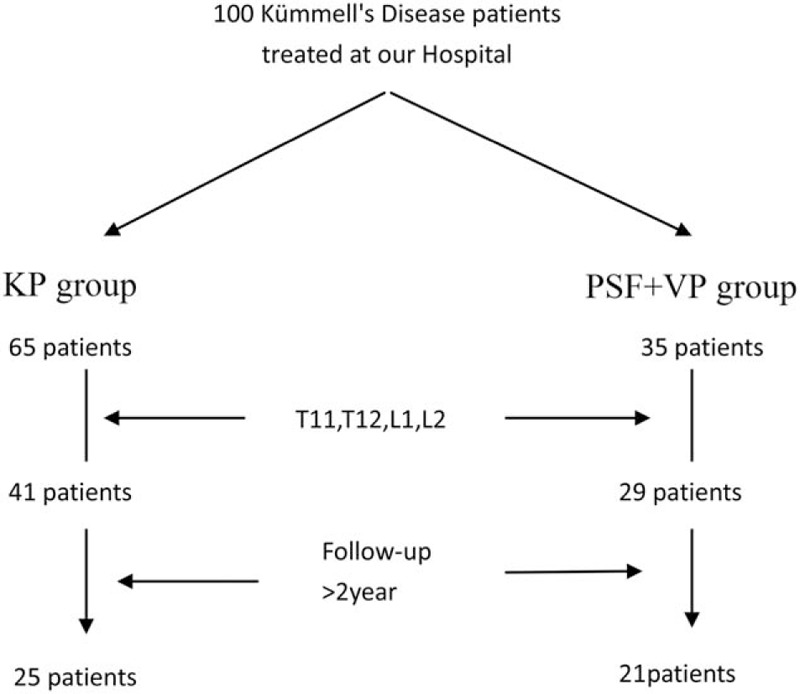
Of the 100 patients included for analysis, 65 underwent percutaneous kyphoplasty (KP group) and 35 underwent posterior spinal fixation with vertebroplasty (PSF+VP group). The incidence of KD was more common in the thoracolumbar spine; thus, the study was restrict vertebral segments T11 to L2. There were 41 patients in the KP group and 29 in the PSF+VP group. When limited to patients with more than 2 years of follow-up, there are 25 patients in the KP group and 21 in the PSF+VP group.

The patients were assigned into 2 groups. Among them, 65 patients underwent KP; the residual 35 cases obtained posterior spinal fixation with vertebroplasty were included in PSF+VP group. The incidence of KD was more common in the thoracolumbar spine; thus, the study was located at thoracolumbar region. As a result, there were 41 patients in the KP group and 29 in the PSF+VP group. When limited to patients with more than 2 years of follow-up, there are 25 patients in the KP group and 21 in the PSF+VP group (Fig. 1). There were no significant differences in patient age, disease duration, or the length of follow-up between the 2 groups (Table 1).

**Table 1:**
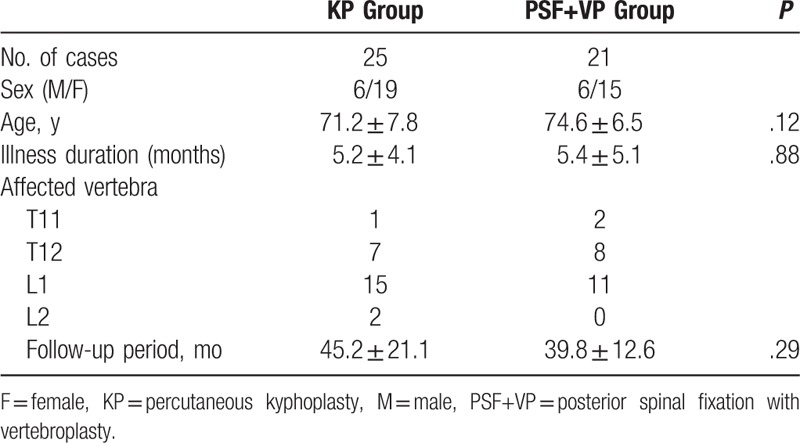
Summary of patient background data.

### 2.2 Percutaneous kyphoplasty (KP)

**Figure 2:**
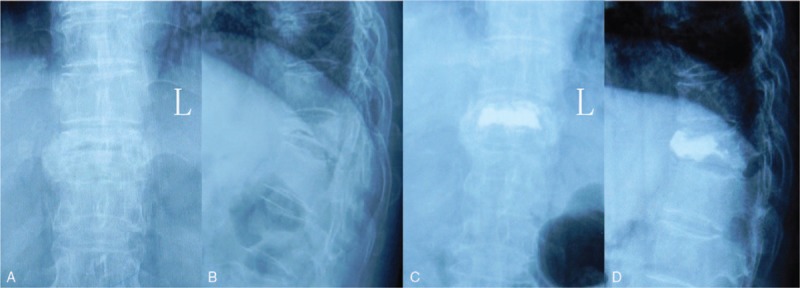
Representative case of percutaneous kyphoplasty. Preoperative posteroanterior (A) and lateral (B) radiographs of a 70-year-old female who underwent KP for KD at T11. The preoperative local vertebral cobb angle was 20°, which was corrected to 12° after surgery. Postoperative posteroanterior (C) and lateral (D) radiographs 2 years after surgery. Loss of correction was 5°.

Under extrapedicular infiltration anesthesia, each patient was placed in the prone position on a radiolucent operating table. A C-arm was used to provide simultaneous anteroposterior and lateral image of the injured vertebrae. A Jamshidi Bone Marrow Biopsy Needle (Tecres S. p. A., Sommacampagna, Italy) was introduced into the pedicle to the cleft of the vertebral body. Then, a guide wire was inserted into the Jamshidi needle and the needle was removed. Several dilating cannulas were passed over the guide wire until an appropriate working cannula was established. Then, the balloon was deflated and removed, A little bone cement (PMMA, Tecres S. p. A., Sommacampagna, Italy) was introduced into the vertebral body via the cannula. We use C-arm to evaluate the mount of the bone cement. And, we continued to perform the process of cement infiltration until the satisfactory cement distribution was confirmed under fluoroscopy. The cannula was removed afterward and the incision was closed (Fig. 2).

### 2.3 Posterior spinal fixation with vertebroplasty (PSF+VP)

**Figure 3:**
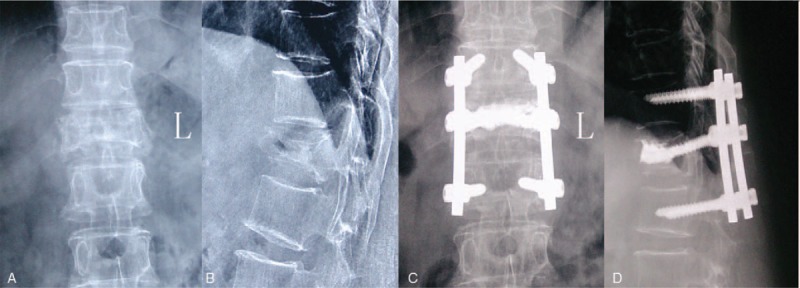
Representative case of posterior spinal fixation with vertebroplasty. Preoperative posteroanterior (A) and lateral (B) radiographs of a 69-year-old-female who underwent PSF+VP surgery for KD at T12. The preoperative local vertebral Cobb angle was 19°, which was corrected to 10° after surgery. Postoperative posteroanterior (C) and lateral (D) radiographs taken 2 years after surgery. Loss of correction was 1°.

A standard posterior technique was performed in which each patient was placed in the prone position on a radiolucent operating table. Postural restoration on the table was performed in order to correct kyphotic deformities without overcorrection. A pedicle probe was used to examine the unilateral pedicle of the symptomatic vertebra. A Jamshidi Bone Marrow Biopsy Needle was introduced into the pedicle and to the cleft of the vertebrae. Then, a guide wire was inserted into the Jamshidi needle (Tecres S. p. A., Sommacampagna, Italy) and the needle was removed. Several dilating cannulas were passed over the guide wire until an appropriate working cannula was established. Bone cement (PMMA, Tecres S. p. A., Sommacampagna, Italy) was introduced into the cleft and the pedicle screw was placed into the affected vertebra. Pedicle screws (Beijing Sanyou Intellectual Property Agency Ltd, Beijing, China) were placed into the symptomatic vertebra and adjacent level. Of note, short pedicle screws were placed into the symptomatic vertebra. The satisfactory cement distribution and pedicle screws position was confirmed under fluoroscopy. The incision was closed (Fig. 3).

### 2.4 Postoperative treatment

KP patients lied on their back for 8 hours, the lower limb sensory and motor activities were observed, and the vital signs were monitored. Postoperative X-ray films were performed to observe the distribution of the bone cement of the vertebrae the next day after surgery. Two days after surgery, with the protection of braces, recovery activities were allowed to take regularly. PSF+VP patients lied on their back for 8 hours, the lower limb sensory and motor activities were observed, and the vital signs were monitored. Usually, patients in this group required more bed rest. When the volume of blood drainage was less than 50 mL, the drainage tube was removed. Postoperative X-ray films were performed to observe the distribution of the bone cement of the vertebrae and position of the pedicle screw the next day after drainage tube was removed. Patients were permitted to engage in recovery activities with the protection of brace for at least 3 months. All patients used routine anti-osteoporosis treatments.

### 2.5 Evaluation

**Figure 4:**
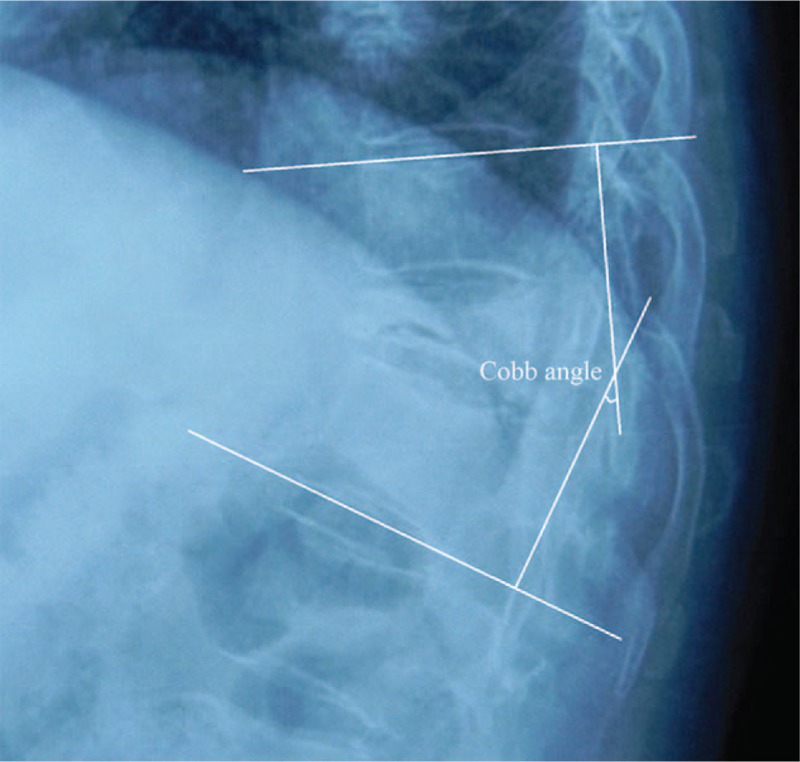
Kyphotic Cobb angle (CA): the angle between the upper and lower endplates of vertebral body with the most serious kyphosis approached up or down by vertebral fracture. CA = Cobb angle.

We established contact with patients at 1 year postoperatively, postoperatively, and the last follow-up by telephonic follow-up. Back pain was assessed by visual analog scale (VAS) and Oswestry Disability Index (ODI). We reviewed the lateral X-ray, and measured the sagittal Cobb angle on the lateral X-ray images preoperatively, immediate postoperatively, 1 year postoperatively, 2 year postoperatively, and last follow-up (Fig. 4). The operation time, bleeding volume, and complications were also compared between the 2 groups.

### 2.6 Statistical analysis

All data were expressed as mean ± SD. Age, illness duration, follow-up period were compared using Chi-square tests. Pre- and postoperative measurement data including VAS score, ODI, and local kyphotic angle were compared using a paired Student *t* test. *P* < .05 was considered to indicate statistical significant difference. Statistical analyses were conducted using SPSS version 17 (SPSS Inc, Chicago, IL).

## 3 Results

The surgical results of KP and posterior spinal fixation with vertebroplasty (PSF+VP) for treating KD achieved satisfactory outcome with regard to VAS score, ODI score, and Cobb angle. However, the mean operation time was significantly longer, and blood loss significantly greater, in the PSF+VP group. All in all, we prefer KP for the treatment of KD with monosegment lesion and without neurological deficit.

### 3.1 Comparisons of operation time, bleeding volume

The mean operation time was 43.2 ± 21.8 minutes in the KP group and 230.6 ± 87.1 minutes in the PSF+VP group; the mean bleeding volume was 5.3 ± 3.1 mL in the KP group and 215.0 ± 170.2 mL in the PSF+VP group. As these figures make clear, the mean operation time was significantly longer, and blood loss significantly greater, in the PSF+VP group.

### 3.2 VAS score

**Figure 5:**
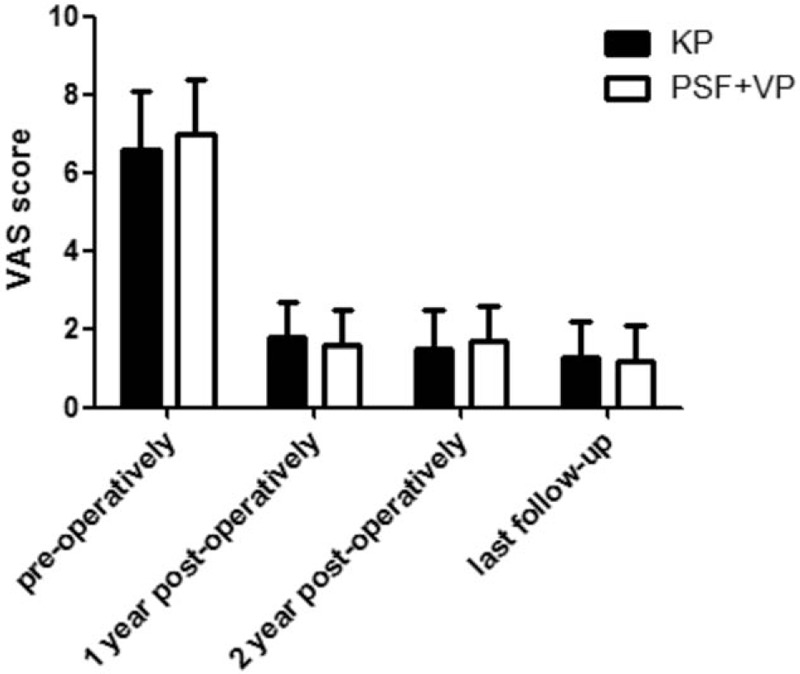
Mean ROM (±SD) for this group in the visual analog scale (VAS) as evaluated and compared between KP group and PSF+VP group preoperatively, 1 year postoperatively, 2 year postoperatively, and at last follow-up. An asterisk (^∗^) indicates a significant difference between the corresponding groups (as indicated by the horizontal bar) at *P* < .05.

In the KP group, mean VAS scores were 6.6 ± 1.5 pre-operatively, 1.8 ± 0.9 at 1 year postoperatively, 1.5 ± 1.0 at 2 year postoperatively, and 1.3 ± 0.9 at the last follow-up (Fig. 5). In the PSF+VP group, mean VAS scores were 7.0 ± 1.4 pre-operatively, 1.6 ± 0.9 at 1 year postoperatively, 1.7 ± 0.9 at 2 year postoperatively, and 1.2 ± 0.9 at the last follow-up. There was no significant difference between the 2 groups.

### 3.3 ODI score

**Figure 6:**
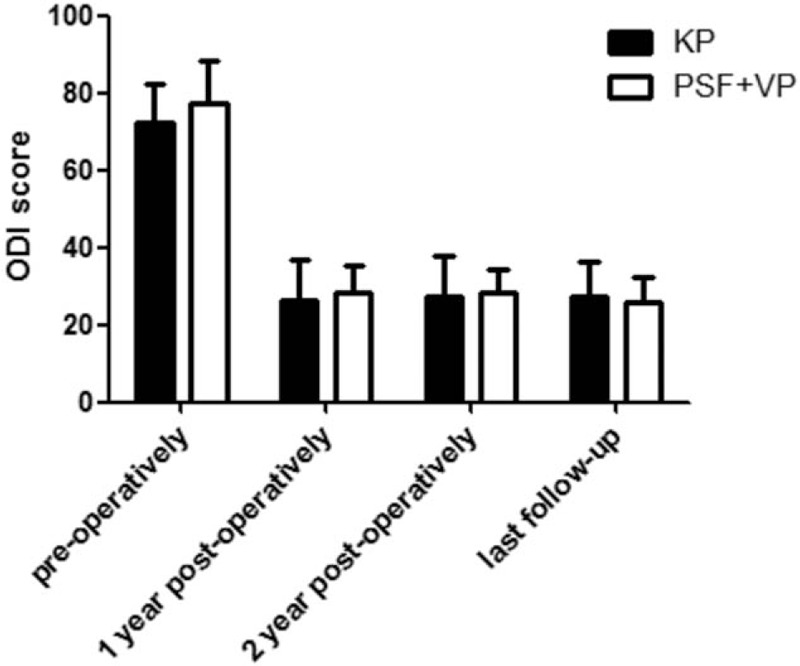
Mean ROM (±SD) for this group in the Oswestry disability index (ODI) was evaluated and compared between KP group and PSF+VP group preoperatively, 1 year postoperatively, 2 year postoperatively, and at last follow-up. An asterisk (^∗^) indicates a significant difference between the corresponding groups (as indicated by the horizontal bar) at *P* < .05.

In the KP group, mean ODI scores were 72.5 ± 10.0 preoperatively, 26.1 ± 10.6 at 1 year postoperatively, 27.1 ± 10.5 at 2 year postoperatively, and 27.2 ± 9.0 at the last follow-up (Fig. 6). In the PSF+VP group, mean VAS scores were 77.5 ± 10.6 pre-operatively, 28.1 ± 7.4 at 1 year postoperatively, 28.2 ± 6.1 at 2 year postoperatively, and 26.0 ± 6.3 at the last follow-up. There was no significant difference between the 2 groups.

### 3.4 Cobb angle

**Figure 7:**
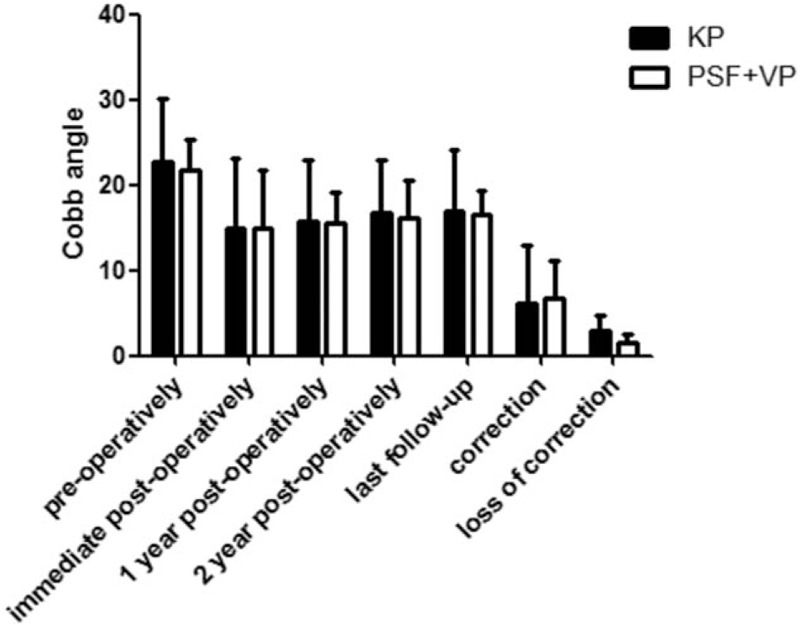
Mean ROM (±SD) for this group in the vertebral Cobb angle was evaluated and compared between the 2 groups preoperatively, immediate postoperatively, 1 year postoperatively, 2 year postoperatively, at last follow-up, correction, and loss of correction. An asterisk (^∗^) indicates a significant difference between the corresponding groups (as indicated by the horizontal bar) at *P* < .05.

In the KP group, mean local kyphotic angles were 22.8 ± 7.4 preoperatively, 14.9 ± 8.2 immediate postoperatively, 15.7 ± 7.2 at 1 year postoperatively, 16.8 ± 6.2 at 2 years postoperatively, and 17.0 ± 7.2 at the final follow-up (Fig. 7). Immediately after surgery, 6.2 ± 6.8 mean correction from preoperative levels was observed for kyphosis, but 3.0 ± 1.8 mean loss of correction was observed. In the PSF+VP group, mean local kyphotic angles were 21.7 ± 3.6 preoperatively, 15.0 ± 6.7 immediate postoperatively, 15.6 ± 3.6 at 1 year postoperatively, 16.2 ± 4.3 at 2 years postoperatively, and 16.5 ± 2.8 at the final follow-up. Immediately after surgery, 6.8 ± 4.3 mean correction from preoperative levels was observed for kyphosis, but 1.6 ± 0.9 loss of correction was observed.

### 3.5 Postoperative complication

As for the postoperative complications, in the KP group, cement leakage out of the vertebrae was observed in 2 patients (8.0%). Both patients have no symptom. In the PSF+VP group, cement leakage out of the vertebrae was observed in 1 patient (4.8%). The patient got symptomless. The superficial wound infection was observed in 2 patients (9.5%). All patients recovered without any adverse effects.

## 4 Discussion

The pathogenesis of KD remains controversial. It has been mainly theorized to involve fracture nonunion, avascular necrosis, or pseudarthrosis. Each of these theories can explain the cause of KD to a certain aspect.^[16–19]^ The causes of pain in KD are also diversified. It is mainly due to micro-movement of the vertebral fracture. Elimination of the micro-fractures and vertebral stabilization can result in effective pain reduction and satisfactory clinical outcomes.^[20]^

KD patients with neurological symptoms or those without neurological symptoms need different treatment strategies. For patients with neurological symptoms, the objective of surgery is to decompress the spinal canal and maintain spinal stability. The surgical methods usually include anterior, posterior, or combined anterior and posterior approaches. For patients without neurological symptoms, the aim is to eliminate micro motion at the fracture sites. Some articles reported that PVP or PKP treat for KD without neurological symptoms achieved good clinical result. These minimally invasive procedures were important for older patients with comorbidities. Particularly, it is good for postoperative rehabilitation.^[21,22]^ Just like previous studies, we also found significant pain reduction and good clinical results following kyphoplasty. After kyphoplasty, elimination of microscopic motion at the fracture site and stabilization of the fractured vertebrae is generally believed to result in pain relief. Other important mechanisms of pain relief include a thermal neurolytic or chemical effect of bone cement.^[23,24]^

However, KD is different from common osteoporotic vertebral compression fractures (OVCFs) and described by sclerosis and bone absorption. KD patients were diagnosed in the late stage, usually with severe backache and kyphosis. The type of cement filling is quite particular. For the majority of patients, bone cement augmentation acts as a supporting block, for the lack of cement diffusion into cancellous bone, the holding-on effect between cement and bone is insufficient. However, it is showed that 2 cases happening delayed cement displacement following PVP or PKP alone for KD without neurological deficits.^[14,25]^ Because of this, the PSF+VP appeared as a kind of measure with more stability.

The relationship between the severity of back pain and hyper kyphosis in OVCF was another controversial issue. Some authors found that vertebral deformities cause substantial pain.^[26]^ However, the other authors investigated reported that symptoms were related to vertebral deformities.^[27]^ On the contrary, another previous study found that the improvement of VAS score had no correlation with the improvement of local kyphotic angle.^[24]^

Our study did not find an association between backache relief and kyphosis correction. The VAS score and ODI score decreased significantly, and local kyphotic angle also improved significantly after kyphoplasty. However, the improvement of the VAS score and ODI score had no significant correlation with the improvement of local kyphotic angle. This suggests that it might be unrelated between the severity of back pain and the severity of the kyphosis. This suggests that correction of local kyphosis might not be the main factor in pain reduction.

Although the 2 kyphosis angles were inconsistent, the 2 groups both attain good clinical result. In our study, the pain from KD closely related to the micro movement of the fracture broken end. Both groups stabilized the broken ends. There was no bone cement displacement.

Up to now, only case reports and small series of patients have been reported. In 2011, Lee et al^[15]^ reported satisfactory results for 10 patients with KD treated by vertebroplasty combined with short segmental fixation. However, the sample of the patients are relatively small.

In our study, both KP and PSF+VP achieved satisfactory outcome for KD with regard to backache relief and kyphosis correction. There was no statistical difference between the 2 groups with regard to pain reduction and radiographic outcome. Although both groups had cement leakage out of the vertebrae, superficial wound infection happened in PSF+VP group. All patients recovered without any adverse effects, suggesting both kinds of procedures were safe and effective. Comparatively, the PSF + VP group required a significantly longer operation time, bleeding volume, and hospital stay at greater costs.

Compared with KP, PSF+VP show the advantages in simplifying surgical procedure, saving operation time, minimizing trauma, and controlling blood loss, and the relative risk of postoperative infection is low. PSF+VP group is good than KP group for the loss of kyphosis.

However, that correction of local kyphosis might not be the main factor in pain reduction.

The strengths of our study are listed as follows. First, our study has a relatively large sample about 46 patients with KD. Second, we evaluate the patients with iconography examination and the improvement of symptoms. Third, the follow-up time is relatively long. The limitations of this study were a retrospective analysis and both groups included old patients with comorbidities, the influence of which was not analyzed. Despite these mentioned limitations, this study assessed and compared the clinical outcome of 2 commonly used procedures for a relatively difficult disease, and provided useful information.

We recommend KP for the treatment of KD. Especially for old age with poor constitution, this conclusion will provide orthopedist an effective way for treatment of KD. The future direction of our study is to make a prospective study and bring into more patients to our study.

## 5 Conclusion

This study shows that both treatments KP and PSF+VP for KD can be safe and effective. KP resulted in a shorter surgical duration, less blood loss, and fewer postoperative complications. Therefore, we recommend KP for the treatment of KD.
